# Integrated machine learning and bioinformatic analyses used to construct a copper-induced cell death-related classifier for prognosis and immunotherapeutic response of hepatocellular carcinoma patients

**DOI:** 10.3389/fphar.2023.1188725

**Published:** 2023-05-17

**Authors:** Shuai Zhao, Shuxian Chen, Wangrui Liu, Shiyin Wei, Xinrui Wu, Dan Cui, Lifeng Jiang, Siyu Chen, Jian Wang

**Affiliations:** ^1^ Department of Transplantation, Xinhua Hospital affiliated to Shanghai Jiao Tong University School of Medicine, Shanghai, China; ^2^ Department of Oncology, Xinhua Hospital affiliated to Shanghai Jiao Tong University School of Medicine, Shanghai, China; ^3^ Department of Interventional Oncology, Renji Hospital, Shanghai Jiao Tong University School of Medicine, Shanghai, China; ^4^ Affiliated Hospital of Youjiang Medical University for Nationalities, Baise, China; ^5^ Department of Urology, Renji Hospital, Shanghai Jiao Tong University School of Medicine, Shanghai, China; ^6^ Department of Gastroenterology, The Affiliated Changzhou No.2 People’s Hospital of Nanjing Medical University, Changzhou, Jiangsu, China

**Keywords:** copper induces cell death, tumor microenvironment, mutation burden, immunotherapy, multi-omics study

## Abstract

**Background:** Copper as phytonutrient has powerful activity against health diseases. A newly discovered mechanism of cell death that affects energy metabolism by copper (“cuproptosis”) can induce multiple cuproptosis-related genes. Hepatocellular carcinoma (HCC) is a poorly prognosed widespread cancer having danger of advanced metastasis. Therefore, earlier diagnosis followed by the specific targeted therapy are required for improved prognosis. The work herein constructed scoring system built on ten cuproptosis-related genes (CRGs) to predict progression of tumor and metastasis more accurately and test patient reaction toward immunotherapy.

**Methods:** A comprehensive assessment of cuproptosis patterns in HCC samples from two databases and a real-world cohort was performed on ten CRGs, that were linked to immune cell infiltration signatures of TME (tumor microenvironment). Risk signatures were created for quantifying effect of cuproptosis on HCC, and the effects of related genes on cellular function of HCC were investigated, in addition to the effects of immunotherapy and targeted therapy drugs.

**Results:** Two distinct cuproptosis-associated mutational patterns were identified, with distinct immune cell infiltration characteristics and survival likelihood. Studies have shown that assessment of cuproptosis-induced tumor mutational patterns can help predict tumor stage, phenotype, stromal activity, genetic diversity, and patient prognosis. High risk scores are characterized by lower survival and worse treatment with anti-PD-L1/CTAL4 immunotherapy and first-line targeted drugs. Cytological functional assays show that CDKN2A and GLS promote proliferation, migration and inhibit copper-dependent death of HCC cells.

**Conclusion:** HCC patients with high-risk scores exhibit significant treatment disadvantage and survival rates. Cuproptosis plays a non-negligible role in the development of HCC. Quantifying cuproptosis-related designs of tumors will aid in phenotypic categorization, leading to efficient personalized and targeted therapeutics and precise prediction of prognosis and metastasis.

## 1 Introduction

HCC is the widespread primary liver cancer and ranks among the top regarding its occurrence and mortality. It has thus great negative impact on society (Cabasag et al., 2022). Compared with most solid cancers, HCC cases and HCC-related mortalities have escalated in past few decades (Siegel et al., 2016). HCC is healed through systemic therapy, liver-directed therapy, liver transplantation, and surgical resection. Of the mentioned options, only liver transplantation, and surgical resection are regarded as potential treatments. However, <20% patients are eligible for potentially curative treatments (Giannini et al., 2015). Systemic chemotherapy is not routinely used because HCC, unlike some other solid cancers, is considered to be chemoresistant with overexpressed dihydropyridine dehydrogenase, the multidrug resistance gene MDR-1, and P-glycoprotein gene product (Kim et al., 2017). To date, sorafenib continues to be only FDA-certified drug for HCC, but its effects are not satisfactory.

The complicated contact of effector molecules with immune cells in tumor microenvironment surrounding HCC changes immune system and can promote or inhibit HCC progress (Oura et al., 2021). The elucidation of multimodal processes of immunotherapies may improve earlier and advanced HCC effects. Targeted therapies for canonical tumor-associated pathways in tumor microenvironment have been approved or are in trials at clinical levels for multiple cancers, however proof for the therapy effects for HCC is inadequate (Bray et al., 2018). Lenvatinib proved to be as effective as sorafenib, as lenvatinib-treated patients had similar overall survival (OS) rates compared with sorafenib-treated patients. Additionally, lenvatinib showed significant improvement in progression-free survival (PFS), responses, and disease control rates. The second line treatment of unresectable HCC which is intolerant toward lenvatinib or sorafenib includes sunitinib tyrosine kinases, cabozantinib, regorafenib, and related pathways (Llovet et al., 2021). However, these molecularly targeted therapies exhibit cytostatic properties, drug resistance, and severe adversities, that hamper therapeutic benefits and patient tolerability. Today, immunotherapy has received more attention and has been promoted as a first line cure for diverse cancer types. Specific subtype of membrane-bound molecules termed as immune checkpoints, regulate cancer immune evasion by promoting T cell exhaustion and blocking T cell activation. CTLA-4 and PD-1/PD-L1 are primary targets for immune checkpoint inhibitors (ICIs), some of which are currently approved or in clinical trials for HCC (Zhang et al., 2017; Seidel et al., 2018). Because of tumor heterogeneity, TME alteration, development of drug resistance, hypervascularity, hypoxia, and severe side effects, treatment of HCC with ICIs or ACT alone failed to achieve primary clinical endpoints, including a reduction in tumor size and antitumor response (Altekruse et al., 2014; Zhao et al., 2021a). Therefore, developing markers is crucial which may help physicians to choose the cases for benefitting from the treatment effects prior to or during early treatment (Zhao et al., 2021b). This also boosts therapeutic benefits of combinatorial therapy along with other molecularly targeted drugs.

Today, crops are under severe abiotic stress in farmland, and heavy metal toxicity is the most important limiting factor, which not only reduces the growth of plants, but also deteriorates the quality of food. Copper, a transition metal that is found in low concentrations in biological tissues but is essential for life, has also been identified as a phytonutrient. Copper ionophores can transport copper into cells, and multiple lines of evidence suggest that the process of inducing cell death through copper ionophores implies intracellular copper being accumulated rather than influence of small molecular chaperones. The experiments on relationship of structure with function have shown that modifications eliminating copper binding capacity of these compounds cause loss of cell death (Kim et al., 2008). However, for cancer, utilization of copper-related therapies has been effective to lesser extent (Lutsenko, 2010; Ge et al., 2022): Multiple clinical trials have tested copper ionophores, however these tests were conducted without using biomarkers in appropriate patient populations and not understanding the mechanism of action of drug. Clinical trials of elexicrolol in melanoma patients have shown that this drug is more effective for patients having lower plasma lactate dehydrogenase (LDH) amounts (Tsvetkov et al., 2019). The latest cutting-edge research has found that cells enduring mitochondrial respiration are especially responsive to copper ionophores, which leads to a new manner of cell mortality. Cell death induced by copper occurs through targeting the fatty acylated tricarboxylic acid cycle (TCA) cycle proteins in a process called “cuproptosis” (Tsvetkov et al., 2022). This latest finding describes that copper ionophore therapy must be targeted through this metabolic profile. Therefore, clinical trials in future should be conducted on biomarker-driven copper ionophores.

In the present study, we performed thorough assessment of expressions of cuproptosis-related genes and characterized tumor microenvironment. Employing expressions of cuproptosis-related genes, HCC specimens were grouped to three cuproptosis-related clusters. Samples were then divided to 2 distinct gene clusters on the basis of the differentially expressed genes (DEGs) discovered in three clusters. Subsequently, 2 core genes, CDKN2A and GLS, were detected by employing multivariate COX regression evaluation and least absolute shrinkage and selection operator (LASSO). With this, cuproptosis-related prognostic model and scoring system were constructed, allowing predictive prognosis and survival analysis of both database and real-world patients, as well as a detailed assessment of patient response to immunotherapy.

## 2 Materials and methods

### 2.1 Research data

Gene expression quantification, demographic characteristics and clinical parameters among HCC patients were thoroughly searched and then downloaded from TCGA (The Cancer Genome Atlas). Patients were excluded according to following criteria: 1) erroneous survival information, such as survival days smaller than 0; 2) lack of survival period.

### 2.2 The cuproptosis-related genes unsupervised clustering analysis

Total of ten CRGs were abstracted from “Targeting lipoylated TCA cycle proteins with copperring induces cell death” (Tsvetkov et al., 2022). In order to determine the underlying biomarkers, unsupervised clustering analysis was carried out for the identification of distinct cuproptosis-related clusters. Furthermore, to characterize potential biological pathways of CRGs, gene set variation analysis (GSVA) method was firstly applied to estimate enrichment scores against *c2.cp.kegg.v7.2* gene set using package *GSVA*. Next, gene ontology (GO) enrichment analysis was utilized to interpret gene sets via *clusterProfiler*.

### 2.3 Clinical annotation and functional of the cuproptosis-clusters

Clinicopathological characteristics of HCC patients and relationships between CRG clusters and recurrence free survival were investigated. The prognosis prediction efficacy of the molecular subtypes was also determined. Demographic and clinical parameters, including age, gender, ethnicity, residual tumor, TNM stage, primary therapy outcome, grade, and tumor stage were described in detail and included in the following models as covariates. The overall survivals between patients assigned to different cuproptosis-related clusters by packages, *survminer* and *survival*, were compared through Kaplan-Meier survival analysis. Disease ontology (DO) and Kyoto Encyclopedia of Genes and Genomes (KEGG) algorithms were performed for identifying biochemical pathways and biomedical ontologies.

### 2.4 Establishment of the cuproptosis-related prognostic risk signature

In order to explore risk signature to predict the prognosis of HCC patients, the popular method LASSO regression was firstly considered to recognize DEGs between cuproptosis-related clusters. Next, multivariate Cox proportional hazards regressions were performed for evaluating associations for DEGs with survival period of HCC patients, and only significant DEGs were remained. Then, HCC patients were classified into distinct clusters via unsupervised clustering analysis. Patients from ICGC and TCGA database were merged together and then randomly partitioned to testing and training sets at 1:1 ratio. In training set, Cox proportional hazard regressions with covariates adjusted were carried out to identify candidate genes for risk signature. LASSO algorithm was additionally performed to combat the risk of overfitting. Packages glmnet and survival were used in the analysis.

### 2.5 PD-1/L1 expression analysis and somatic mutation

Fractions of immune cells were quantified among HCC patients in both high- and low-risk classes using CIBERSORT algorithm. Correlations between three selected genes and immune cells proportions were explored. Microsatellite instability (MSI) status was compared between different risk groups. ESTIMATE method was conducted for assessing proportion of stromal and immune cells for tumors and evaluate tumor purity by estimate. Then, tumor mutation burden (TMB) score of HCC patients was computed and then utilized for comparison of somatic mutations among different risk groups. In addition, expression levels of certain genes related to immune checkpoints were compared between risk groups.

### 2.6 Nomogram construction

To clearly visualize the risk assessment for each HCC patients, nomogram was built up with above variables involved. Besides, receiver operating characteristic (ROC) curves were drawn for suggesting survival probability in 1, 3, and 5 years and evaluate discriminant efficacy of models.

### 2.7 Cell culture and transfection

Human-derived HCC cell lines HepG2 and Hep3B were procured from Shanghai Institutes of Biological Sciences (China). HepG2 and Hep3B were cultured in medium of RPMI 1640 (Gibco, United States) supplement by 1% penicillin-streptomycin (Gibco, United States) and 10% fetal bovine serum (Gibco, United States). Knockdown of CDKN2A gene was carried out by transient silencing using the following sequences: siRNA1: 5′ - GAT CAT CAG TCA CCG AAG G - 3′; siRNA2: 5′ - AAA CAC CGC TTC TGC CTT T - 3′. In addition, small interfering RNA targeted at GLS gene was obtained, whose sequences were: siRNA1: 5′ - GAU GGA CAG AGG CAU UCU A - 3′, siRNA2: 5′- CCC AGG UUG AAA GAG UGU A -3′. Transfections of cell were made by employing Lipofectamine 3,000 (Invitrogen; United States), with oligonucleotides as control. In 48 h after transfection, cellular RNA and protein were extracted.

### 2.8 Cell migration assays and proliferation

Stably transfected Hep3B and HepG2 were placed at 5 × 104 cells/mL into 96-well plate. Cellular proliferative capacity was tested by Cell Counting Kit-8 (Dojindo, Japan). In each day of the subsequent 6 days, optical density was evaluated at 450 nm using microplate reader (TEAN, Swiss). Besides, in order to study migratory response of Hep3B and HepG2, transwell migratory assay was conducted. Density of cell was standardized as 2 × 105 cells/mL, and 100 μL cell suspension was put in to upper portion. In lower portion, 20% fetal bovine serum medium was put in. After 24 h, Hep3B and HepG2 within lower portion were washed for 15 min in 4% polyoxymethylene, for 30 min, 0.1% crystal violet and deionized water, and then recorded via microscope.

### 2.9 Statistical analysis

R software (version 4.0.0) was employed for all the analyses. Results of *p*-value < 0.05 was statistically important.

## 3 Results

### 3.1 Landscape of cuproptosis-related genetic alterations in HCC

**FIGURE1:**
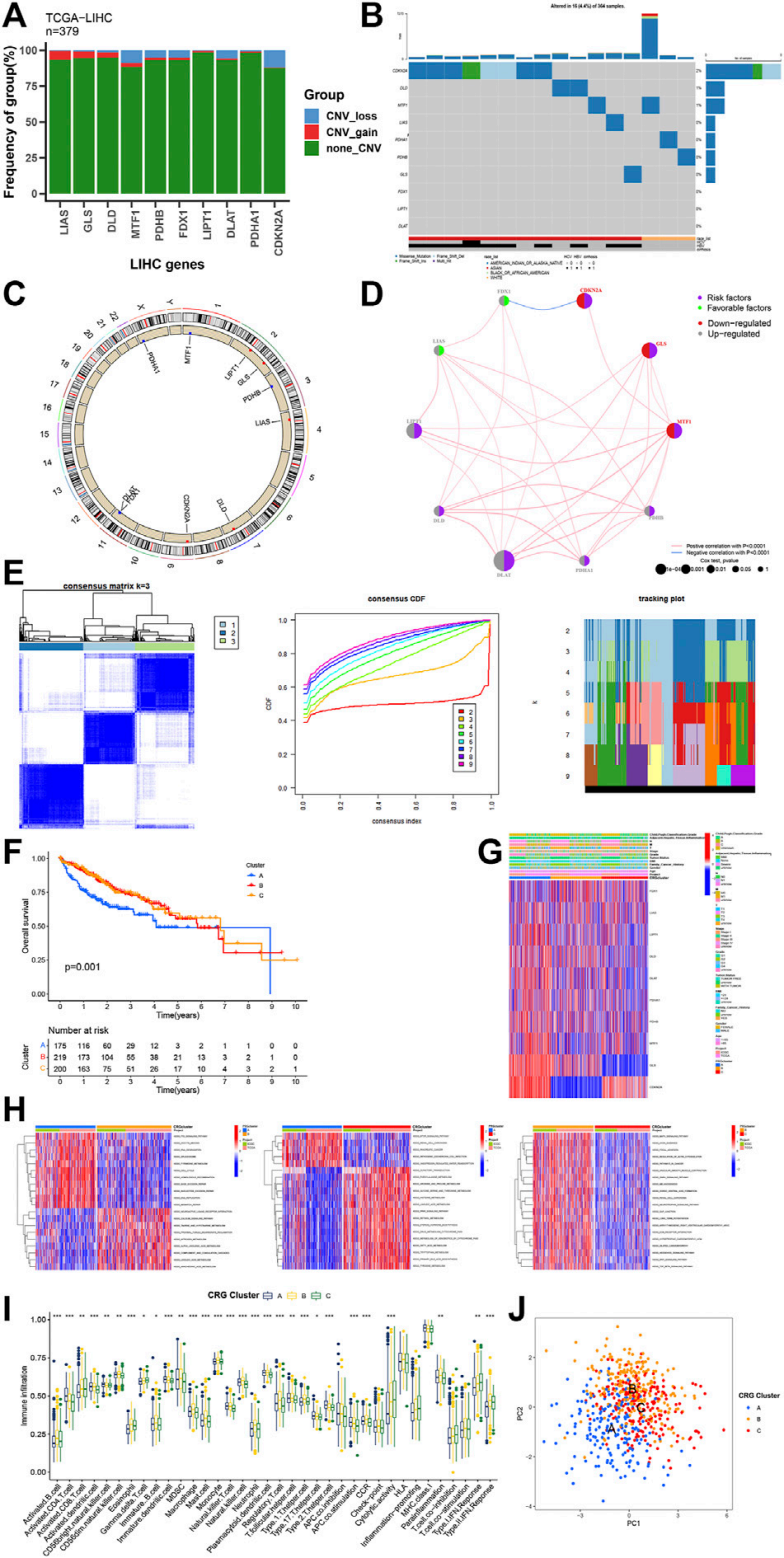
Genetic and transcriptional alterations of CRG. **(A, B)** Mutation frequencies of 10 CRG in TCGA cohort. **(C)** Locations of CNV alterations in CRG on 23 chromosomes. **(D)** The interaction between 10 CRG in HCC. **(E)** Unsupervised clustering analysis heatmap defining three clusters (*k* = 3) and their correlation area. **(F)** Survival analysis of the three CRG clusters. **(G)** Clinicopathological annotation regarding the 10 CRG. **(H)** Heatmap of GSVA enrichment analysis. **(I)** Box plot demonstrating the immune cell-infiltrating characteristics of the three clusters. **(J)** PCA analysis on the three clusters. * *p* < 0.05,**p < 0.01,***p < 0.001.

We first performed comprehensive evaluation of somatic mutations in the cohort of TCGA-HCC among the 10 CRGs included in this work. Outcomes revealed that mutation rates of the CRGs were not very high. CNS-LOSS genes include CDKN2A and MTF1, and CNS-GAIN genes include GLS and LIAS (Figures 1A, B). CDKN2A had maximum frequency of mutation among mutated genes, followed by DLD, MTF1, LIAS, PDHA1, PDHB, GLS, FDX1, LIPT1, and DLAT. Copy number variation (CNV) in these CRGs was then investigated, indicating a prevalent alteration (Figure 1C). The distributions of CNV alterations in 10 CRGs regarding human chromosomes are depicted in Figure 1D. Among them, downregulated CNV was most prominent in genes including CDKN2A, GLS, and MTF1, while upregulated CNV decreases were the most significant in DLAT, LIPT1, and DLD. We combined all patient information in TCGA database and ICGC database to explore the effect of these 10 CRGs on HCC patients (Supplementary Figure S1). We found that these 10 genes had substantial effect on survival rates of HCC patients (*p* less than 0.05), which indicated that cuproptosis had a significant role in promoting the invasion and progression of HCC. Therefore, we conducted further analysis by grouping.

### 3.2 Unsupervised clustering using the cuproptosis-genes in HCC samples

Combining the ICGC and TCGA-HCC cohorts, unsupervised clustering analysis was performed. HCC samples were divided into different molecular subtypes according to CRG expression levels, with three clusters being identified that included cluster A of 175 samples A, cluster B of 219 samples, and cluster C of 200 samples (Figure 1E). Prognostic analysis of the three CRG clusters showed that the survival of those in cluster A was considerably worse than those in other two clusters (Figure 1F, *p* = 0.001). Unsupervised clustering of 10 CRGs in the combined cohort also uncovered three distinct patterns of cuproptosis-related modification, which were both molecularly and clinically significant (Figure 1G). Significantly different KEGG pathway enrichment signatures were noted among three clusters of GSVA enrichment analysis (Figure 1H), which showed that it was mainly associated with strong oncogenic pathways such as P53_SIGNALING_PATHWAY, OOCYTE_MEIOSIS, MTOR_SIGNALING_PATHWAY, _RENAL_CELL_CARCINOMA, MAPK_SIGNALING_PATHWAY, and _AXON_GUIDANCE. Moreover, three cuproptosis-related clusters showed distinct ICICs (Figure 1I). In clusters A and C, the levels of immune cells, like natural killer cells, macrophages, activated CD8^+^ T cells, and activated B cells, were significantly different (*p* < 0.05). Finally, we performed PCA analysis on the three clusters, and it was clear that cluster A was significantly different from clusters B and C (Figure 1J).

### 3.3 GO, KEGG, and DO enrichment analyses in cuproptosis clusters

**FIGURE 2:**
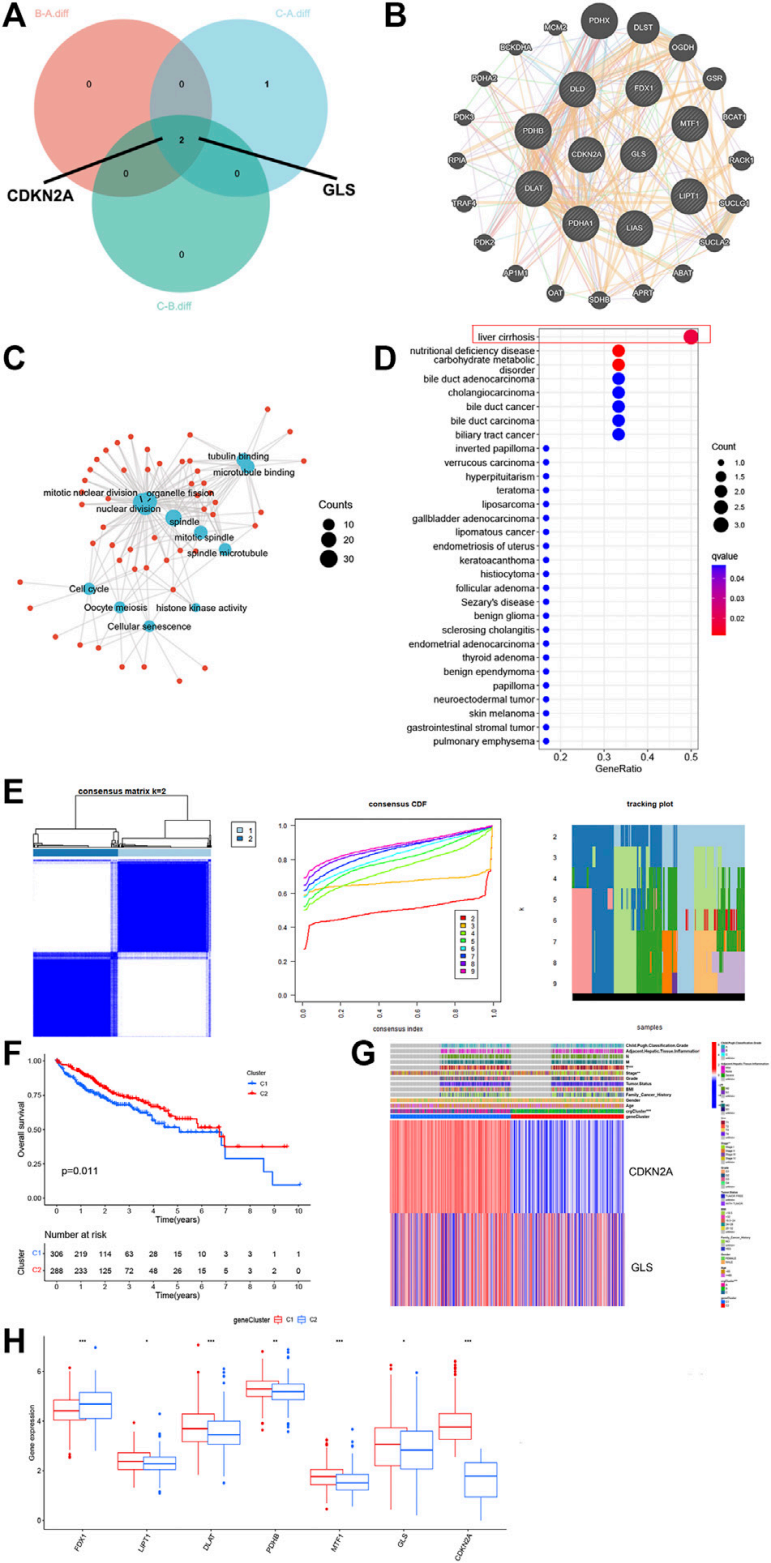
GO, KEGG, DO enrichment and DEG signatures analysis of the CRG. **(A)** Veen plot showed that two genes expressed in all clusters. **(B)** PPI networks for related genes. **(C)** Gene Ontology (GO) enrichment analysis on GO terms enrichment of the CRG. **(D)** Bubble chart of Disease Ontology (DO) enrichment analysis of the CRG. **(E)** Unsupervised clustering analysis heatmap defining two clusters (*k* = 2) and their correlation area. **(F)** Survival analysis of the two gene clusters. **(G)** Clinicopathological annotation regarding the gene clusters. **(H)** Box plot demonstrating the expression levels of the CRGs in the two gene clusters. * *p* < 0.05,**p < 0.01,***p < 0.001.

We analyzed the significant genes of the three CRG clusters, and the Veen plot showed that two genes were significantly expressed in all clusters: CDKN2A and GLS (Figure 2A). For further comprehending traits and biological activities of every copper drooping cluster, we constructed PPI networks for related genes (Figure 2B), then performed GO and KEGG analysis. The CRGs are mainly related to mitotic nuclear division, nuclear division, histones, kinase activity, cell cycle, and cellular senescence (Figure 2C). Next, we performed DO enrichment analysis on the 10 CRGs (Figure 2D). These genes showed significant enrichment in biological processes such as liver cirrhosis, nutritional deficiencies, carbohydrate metabolism disorders, cholangiocarcinoma, and others, that can partially describe elevated recurrence and malignancy rate for HCC. Among them, it can promote the progression of liver cancer by regulating nutrient metabolism and can even be involved in transformation from cirrhosis to liver cancer and the process of cholangiocarcinoma.

### 3.4 DEG signatures in CRG clusters and functional annotation

For further investigating genetic attributes and prospective biological functions of CRG cluster, unsupervised clustering analysis was performed on a total of 614 events from ICGC and TCGA using the previously obtained key genes CDKN2A and GLS. From this analysis, two completely different gene clusters were identified, which were named gene clusters 1 and 2 (Figure 2E). We performed survival analysis on these two gene clusters, clearly finding that the survival of cluster 1 patients was drastically lower than cluster 2 (Figure 2F, *p* < 0.05). Different clinical characteristics between the two clusters can also be noted in the heatmap shown in Figure 2G. We then compared the expression levels of the CRGs in 2 gene clusters (Figure 2H). Significant variations were noticed, most concentrated in gene cluster A, with higher expression of factors unfavorable for HCC survival, such as DLAT, MTF1, GLS, and CDKN2A. These data correspond to the worse prognosis previously found in cluster 1.

### 3.5 Risk signature construction

**FIGURE 3:**
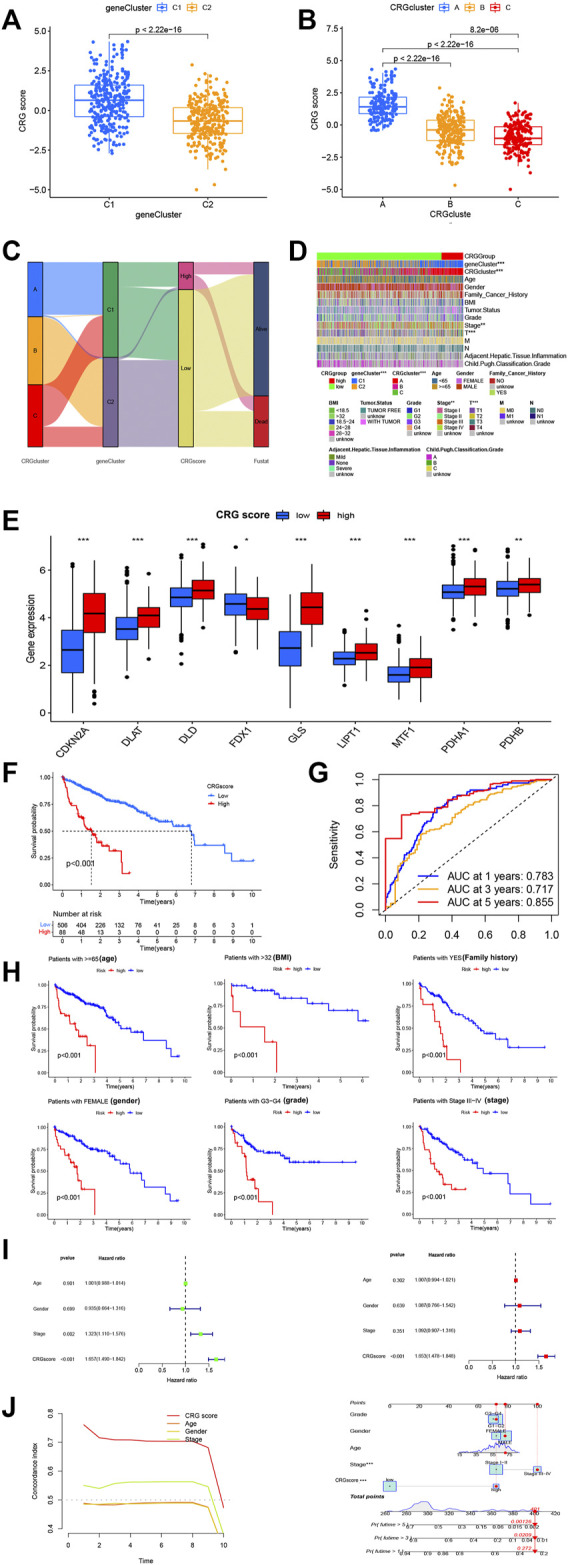
Selection of optimal prognostic signatures and constructure of CRGscore. **(A)** Differences in risk score among gene clusters. **(B)** Differences in risk score among distinct CRG clusters. **(C)** Alluvial diagram showing the changes of CRGclusters, geneClusters, CRG score and future status. **(D)** Heat map regarding the correlation between the CRG score, molecular, genetic classification, prognosis and clinical features. **(E)** Differences in the expression of 10 DEGs among the low-CRGscore group and high-CRGscore groups. **(F)** Kaplan-Meier survival analysis between the high- and low- CRGrisk score groups in cohort. **(G)** ROC curves predicting the sensitivity and specificity of 1-, 3- and 5-year survival according to the CRGsocre. **(H)** Survival analyses conducted on patients with distinct biological background, including age, BMI, family history, gender, grade and stage. **(I)** Univariate COX and Multivariate COX regression analysis in the merged cohort. f. Multivariate COX regression analysis in the merged cohort. **(J)** A nomogram containing the risk score and clinicopathological parameters was established to predict the 1-, 3-, and 5-year survival rates of HCC patients * *p* < 0.05,**p < 0.01,***p < 0.001.

Multivariate COX regression and LASSO analyses were performed, and CDKN2A and GLS were selected as core genes for setting up the risk profiles. Caret R package was utilized to randomly divide all HCC patients (*n* = 614) to 2 groups: a training group (*n* = 307) and test group (*n* = 307). The training group was employed for constructing signature. As per outcomes of multivariate COX regression analysis, patients of risk scores above average were classified as high-risk (*n* = 108), whereas patients with risk scores below average were classified as low-risk (*n* = 506). This scoring approach was used for each HCC sample, and patients categorized to CRG-cluster A and gene cluster 1 were found to have substantially greater risk scores than those in cluster 2 and B/C (Figures 3A, B). Alluvial plots were generated to visualize the patients distribution in three CRG clusters (A-C), two gene clusters (1 and 2), risk score, and state. From this, those among group of low-risk were found with greater probability of survival (Figure 3C). For further investigating relation between risk scores, genetic and molecular classifications, clinical and prognosis characteristics, heatmap was produced for description (Figure 3D). According to the hub genes, PCA scoring was performed to obtain 2 groups: low-CRG and high-CRG. This validates above results in CRG cluster A and gene cluster 1 have worse prognosis than those in the other CRG clusters and gene clusters. To gain further insight into the relationship between risk scores and genetic behavior, we compared and visualized low-CRG and high-CRG groups (Figure 3E). This became apparent that higher the score of the CRG group, the worse the patient’s survival was.

To convincingly validate prognosis power of risk signature, we analyzed survival in combined TCGA and ICGC groups. In general, CRG expression levels were higher in the group of high-CRG. It was hypothesized that higher CRG score was linked to lower survival, and survival decreased with increasing risk score (Figure 3F). To help understand the impact of the two central genes on tumorigenesis and further validate precision and efficacy of risk signature, a combined ROC curve of two was generated (Figure 3G). Analysis of the prognostic classification and prediction efficiency for 1 year (0.783), 3 years (0.717), and 5 years (0.855) showed that AUC values of the risk signature were quite high, confirming that capability of risk signature for predicting upcoming level of HCC patients is quite good. Furthermore, the predictive effect is accurate and time-sustainable, which is not true of many prediction models. The model predicting better over time is invaluable.

In addition, survival analysis was performed on groups with different clinical characteristics, classified according to age, sex, BMI, grade, stage, family cancer history, and more. The results were consistent with the observed lower survival in group of high-risk (*p less than* 0.001). From abovementioned results, it can be tentatively shown that greater risk score is linked to lower survival probability (Figure 3H). Consequent multivariate and univariate COX regression investigations in pooled cohort affirmed that risk signature can serve as independent prognostic model for HCC (Figure 3I). A nomogram with clinicopathological parameters and risk score was developed for predicting 1-year, 3-year, and 5-year rates of survival in HCC patients (Figure 3J). We found that both CRGsocre and stage significantly affected patient survival (*p* less than 0.001), that is in accordance with our earlier works.

### 3.6 TME properties and prospective immunotherapeutic response

**FIGURE 4:**
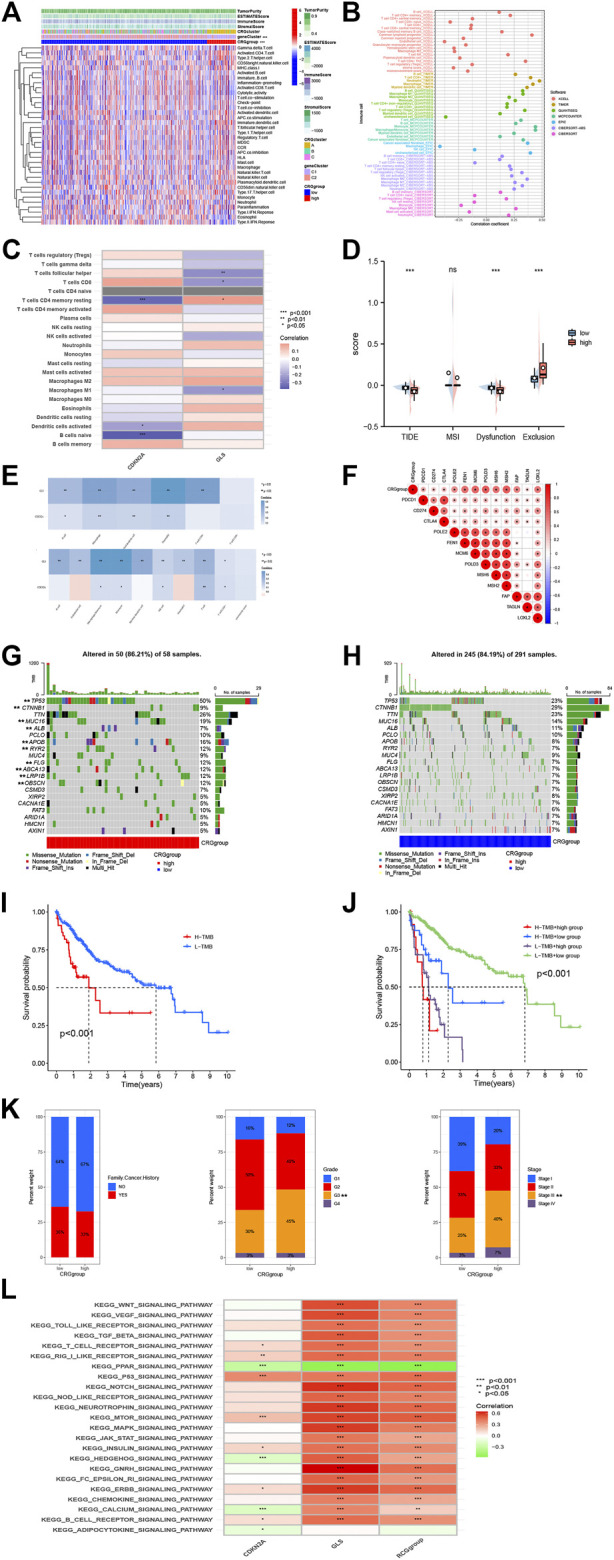
Immune annotation and correlation between HP infection and anti-PD-1/L1, anti-CTLA-4 immunotherapy. **(A)** Relations between tumor purity, ESTIMATE score, immune score, stromal score, and different HCC phenotypes. **(B)** Immune cell-infiltrating characteristics of high- and low-risk CRGgroups and their correlation with risk signature. **(C)** Correlation between the abundance of immune cells and three candidate genes. **(D)** Box plot suggesting the difference of response towards immunotherapy between high- and low-risk CRGscore group. **(E)** A composite risk score was used to assess the association of risk signatures with immune cell numbers in HCC using TIMER and MCPCOUNTER. **(F)** The association of GLS and CDKN2A in HCC with immune checkpoints. **(G, H)** The waterfall plots of tumor somatic mutation constructed by those with low- and high GRCscore. **(I)** Survival analysis on HCC samples with high and low TMB. **(J)** Survival analysis on HCC samples with different TMB and GRCscore. **(K)** Relationships between risk signature and CRGscore. **(L)** GSVA analysis of the KEGG pathway.

Tumor Immunity Estimation Resource (TIMER), CIBERSORT, and other procedures were used to assess link for CRG groups, infiltration of immune cell, envisaged by scatterplots and heatmaps (Figure 4A). They indicated that the CRG group and gene group were considerably correlated with T Cell CD8^+^, T Cell CD4^+^ memory, B Cell, and others (Figures 4B, C). Data from the HCC samples were further analyzed using the TIDE algorithm. As a result, high-CRG group had lower TIDE score, and no substantial variation found between two MSI scores. Regarding dysfunction, high-group is lesser than low-group, while for exclusion, high-group is higher than the low-group. This can be linked to lesser CNV rate of CRGs (Figure 4D). Tumor cell presence rate and tumor purity were significantly higher in high score group as compared to low-CRG group. In addition, a composite risk score was employed for assessing association of risk signatures with immune cell numbers for HCC using TIMER and MCPCOUNTER (Figure 4E). Findings revealed that the CRG score was substantially associated to Neutrophils, macrophages, and myeloid dendritic cells, indicating that these CRGs are also closely related to immune cells. We also analyzed the association of GLS and CDKN2A in HCC with immune checkpoints, which showed are inseparable from multiple immune checkpoints (Figure 4F).

To investigate the relationship between immune infiltration and two CRGs, further analyses were performed. Differences in somatic mutations distribution for low- and high-risk score groups were evaluated by utilizing maftools package (Figures 4G, H, Supplementary Figure S2). To gain further insight into how TMB and risk scores predict outcomes in patients with HCC, additional survival investigations were conducted for CRG groups with various risk and TMB scores. It was confirmed that the CRG group with high TMB had poor survival probability (Figures 4I, J), and high-CRG group survival was much worse compared to low group. Subsequently, correlations between TMB and clinical characteristics, such as family cancer history, grade, and stage, were evaluated (Figure 4K). It was found that CRGsocre was considerably linked with grade and stage (*p* < 0.01), which also indicated that the increase of CRGscore would increase the risk of grade and stage improvement of patients’ tumors, resulting in poor prognosis. Finally, we performed a GSVA analysis of the KEGG pathway, which showed that it was mainly associated with strong oncogenic pathways such as WNT_SIGNALING_PATHWAY, VEGF_SIGNALING_PATHWAY, and TOLL_LIKE_RECEPTOR_SIGNALING_PATHWAY (Figure 4L).

### 3.7 Analysis of immunotherapy and first-line targeted drug therapy

**FIGURE 5:**
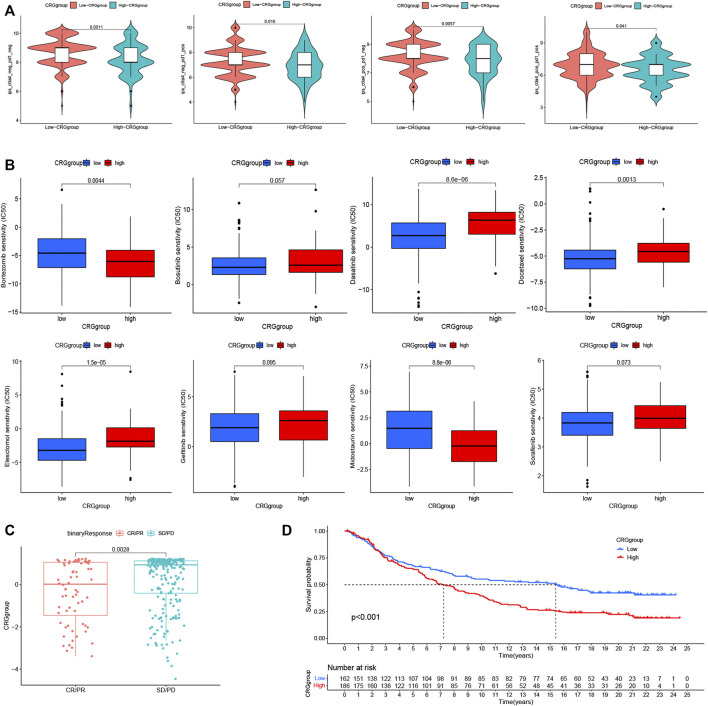
Analysis of immunotherapy and first-line targeted drug therapy. **(A)**. The differences in PD-1 and CTLA-4 treatment between patients in the CRG groups. **(B)**. The analysis of the efficacy of targeted drugs and CRG groups. **(C, D)**. The immunotherapy validation in the IMV210 cohort. * *p* < 0.05,**p < 0.01,***p < 0.001.

We first investigated the differences in PD-1 and CTLA-4 treatment between patients in the CRG groups (Figure 5A). The treatment of CTLA-4 and PD-1 in high-CRG was worse compared to low-CRG group. This indicates that high group has a worse prognosis, and we urgently need to find new treatment methods. We also analyzed the efficacy of targeted drugs (Figure 5B), also finding that most of the targeted drugs have significant differences in the efficacies of the two groups. These data provide guidance for precise medication decisions for patients. Finally, we conducted immunotherapy validation in the IMV210 cohort, and also found that high group patients had worse immunotherapy efficacy and worse survival (Figures 5C, D, *p* < 0.001).

### 3.8 CDKN2A and GLS promote proliferation, migration and suppress copper-dependent death in HCC cells

**FIGURE 6:**
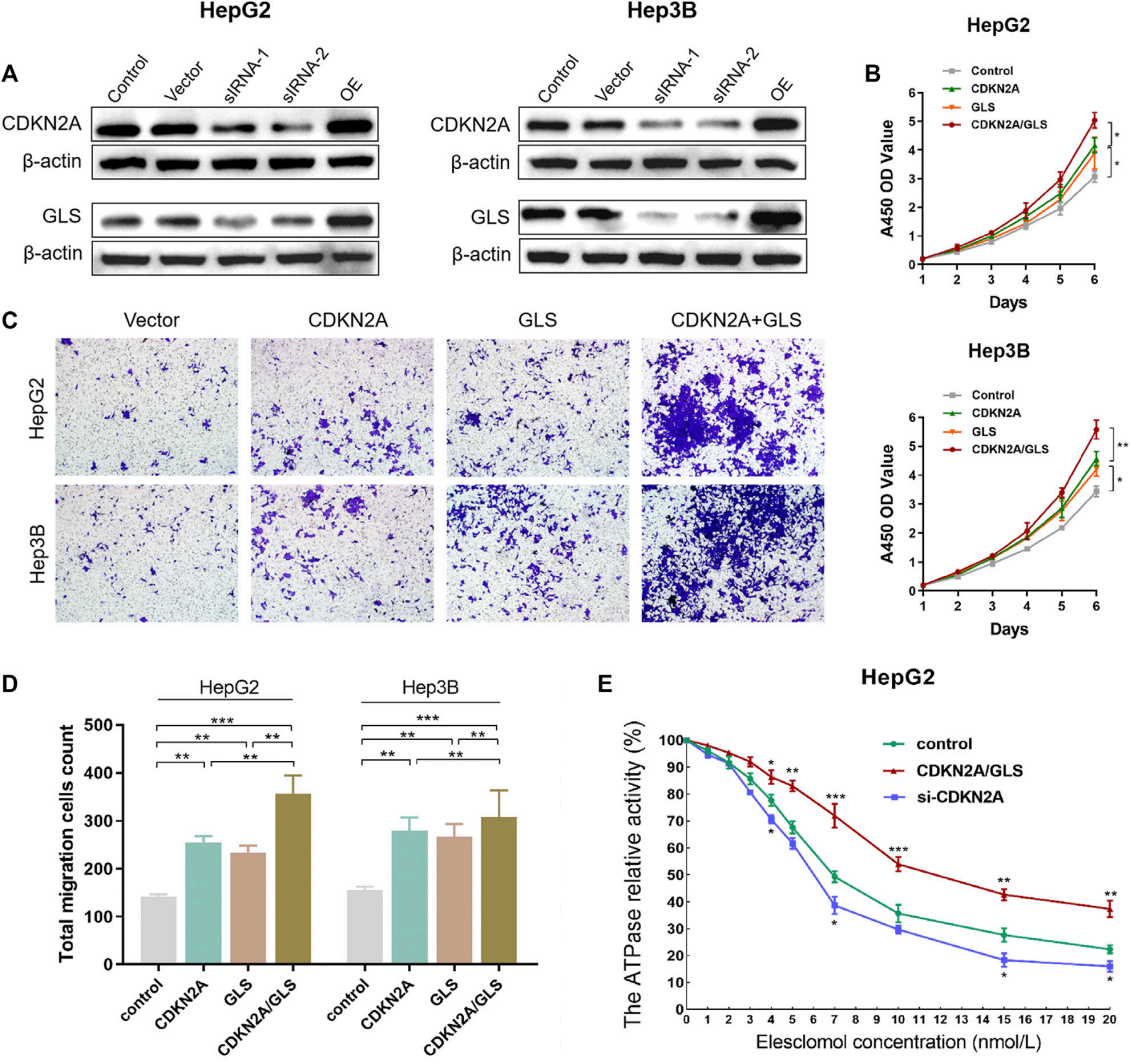
CDKN2A and GLS in HCC cells. **(A)** Downregulated and overexpression of CDKN2A and GLS in HepG2 and Hep3B cells. **(B)** CCK-8 assay, upregulated level of CDKN2A and GLS expression significantly promote the proliferative ability of HCC cells. **(C)**. Transwell cell migration assay analyse the upregulation level of both CDKN2A and GLS expression of HCC cell. **(D, E)**. The ATPase relative activity was prominently elevated in CDKN2A/GLS overexpression group compared with negative control group.

To reveal malignant behaviors of CDKN2A and GLS *in vitro*, we first validation downregulated and overexpression of CDKN2A and GLS in HepG2 and Hep3B cells (Figure 6A). As per outcomes from CCK-8 assay, upregulated levels for CDKN2A and GLS expression promote proliferative capability of HCC cells (Figure 6B). Assay of Transwell cell migration depicted that upregulation of both CDKN2A and GLS expression significantly promote the metastasis ability of HCC cells (Figure 6C). Interestingly, we tested the ATPase relative activity was prominently elevated in CDKN2A/GLS overexpression group compared with negative control group. Overall, the upregulation of CDKN2A and GLS significantly promote proliferation, migration and suppress copper-dependent death in HCC cells (Figures 6D, E).

## 4 Discussion

The unique TME signature of the liver contributes to tumor tolerance and tumorigenesis of fibrosis and chronic inflammation in liver, and immunosuppressive cells are key components promoting HCC growth and invasion (Liu et al., 2020). Presently, different cure solutions of HCC are clinically used for advanced HCC and other cancers, and immunotherapy has more and more concentrated on PD-1 and CTLA-4 monoclonal antibodies which stop the ICI pathway (Yeo et al., 2005). In our study, we found that CRGscore could successfully predict the therapeutic effect of PD-L1/CTAL4 on patients. It was revealed that higher THE CRGscore, worse is immunotherapy effect. This is something that many current tumor markers or tumor models do not have. The positive results of immunosuppressive therapy for HCC have led to shift in cure approach of advanced HCC (Choi et al., 1984). It is considered that findings from current trials at clinical level regarding immunosuppressive therapy of advanced HCC, with different ICIs, will give more understandings to efficient therapy. CRGscore can well predict the efficacy of first-line targeted drugs for HCC patients, which also provides a new idea for further precise treatment. Moreover, developments in RNA and DNA sequencing techniques will give proof of underlying processes of HCC progress for identifying therapeutic targets (Boucher et al., 2002). However, HCC remains one of the diseases with the worst prognosis and requires an entirely new therapeutic strategy. Due to the specificity of HCC, there is an opportunity to tailor novel cell death therapies in HCC.

HCC is a main growing health issue in developed and developing world. Regrettably, for advanced HCC, inadequate systemic cure solutions are existing (Xu et al., 2016). Therefore, in the era of targeted therapy, cytotoxic chemotherapy will have key role in treating advanced HCC, and “cuproptosis,” new way of cell mortality, will bring us new treatment ideas and methods (Zhang et al., 2018). Further studies to understand the complex molecular biology of HCC and to further evaluate effectiveness of combination chemotherapy with molecularly targeted immunotherapy and therapy may improve clinical outcomes. Generally speaking, the “cold and hot” of tumor refers to the hierarchical invasion of tumor cells by relevant immune cells (such as effector T cells) in tumor TME, and the activity of the existing immune system in the microenvironment. More and more evidence shows that the “cold and hot” of tumor will directly determine the effect of immunotherapy. Immunoinflammatory tumor is called “hot tumor,” which is categorized through elevated infiltration of T cells and interferon- γ Increased signal pathway, expression of PD-L1 and greater tumor mutation burden (TMB: higher the TMB, the more new antigens may be generated, and the higher the tumor immunogenicity). Both immune desert and immune rejected tumors may be called “cold tumors”. For immune rejected, lymphocytes of CD8+T are located on the edge of invasion and cannot effectively penetrate tumors; There are no CD8+T lymphocytes in and around immune desert tumors.

In recent years, due to the arbitrary use of pesticides, fungicides, industrial wastewater and wastewater irrigation, copper pollution in agricultural soil is a major problem in sustainable agricultural food production. The growth and yield losses of food crops caused by copper may exceed all other causes of food security and security threats. Many studies have reported the growth inhibition, oxidative damage and antioxidant reaction of Cuinduced on wheat, rice, corn, sunflower, cucumber and other agricultural food crops. The changes of mineral nutrition, photosynthesis, enzyme activity and the reduction of chlorophyll biosynthesis, and the toxic level of copper in crops reduce plant growth and yield. And the content of copper in plants increased significantly. These factors will lead to the further increase of copper absorbed by the human body. Process of copper ionophore-generated cell mortality varies from existing cell mortality pathways, and cure with fatty acids, mitochondrial antioxidants, and mitochondrial function inhibitors, contrasted regarding sensitivity toward ferroptosis-induced GPX4 inhibitor ML162 (Tardito et al., 2011; Nagai et al., 2012; Tardito et al., 2012). Sensitivity has a more significant effect. In addition, inhibitors of mitochondrial pyruvate uptake guided cell death and inhibitors of electron transport chain complexes I and II but had no impact on ferroptosis. Significantly, toxicity of copper was not effected by mitochondrial uncoupler FCCP, describing that mitochondrial respiration, rather than production of ATP, is necessary for copper-induced cell mortality (Cen et al., 2004; Chen et al., 2006). Copper excess helps combination of fatty acylated proteins and Fe-S cluster proteins destabilization, leading to proteotoxic stress and finally cell mortality. Copper-induced cell death is mediated by an ancient mechanism: protein fatty acylation. showed that copper death happens via copper interacting to TCA cycle fatty acylated component, which directs to fatty acylated protein clustering, and iron-sulfur cluster proteins loss, leading to proteotoxic stress and finally cell mortality (Aggarwal and Bhatt, 2018; Raffa et al., 2021). In our study, we conducted correlation analysis according to CRG, and found that cuproptosis involved in related pathways, including p53 Pathway and WNT signal Pathway, are very classic carcinogenic pathways. In addition, Cuproptosis has been associated with cirrhosis and cholangiocarcinoma, which are closely associated with HCC, suggesting that Cuproptosis is likely to be one of the culprits in the progression of HCC.

## 5 Conclusion

The study herein exhibited mutation forms caused by cuproptosis infection contributing to prognosis and immunotherapeutic response. Risk signature, recognized in this work, by quantifying cuproptosis-related mutation forms of HCC, will add to phenotypes category, hence guide towards highly efficient personalized and targeted therapy and more precise prognosis and metastatic prediction.

## Data Availability

The original contributions presented in the study are included in the article/Supplementary Material, further inquiries can be directed to the corresponding author.
