# Supplementary figures and images for: Integrated machine learning and bioinformatic analyses used to construct a copper-induced cell death-related classifier for prognosis and immunotherapeutic response of hepatocellular carcinoma patients
Source: Front Pharmacol. 2023 May 17;14:1188725. doi: 10.3389/fphar.2023.1188725 (PMC10229845; doi:10.3389/fphar.2023.1188725)

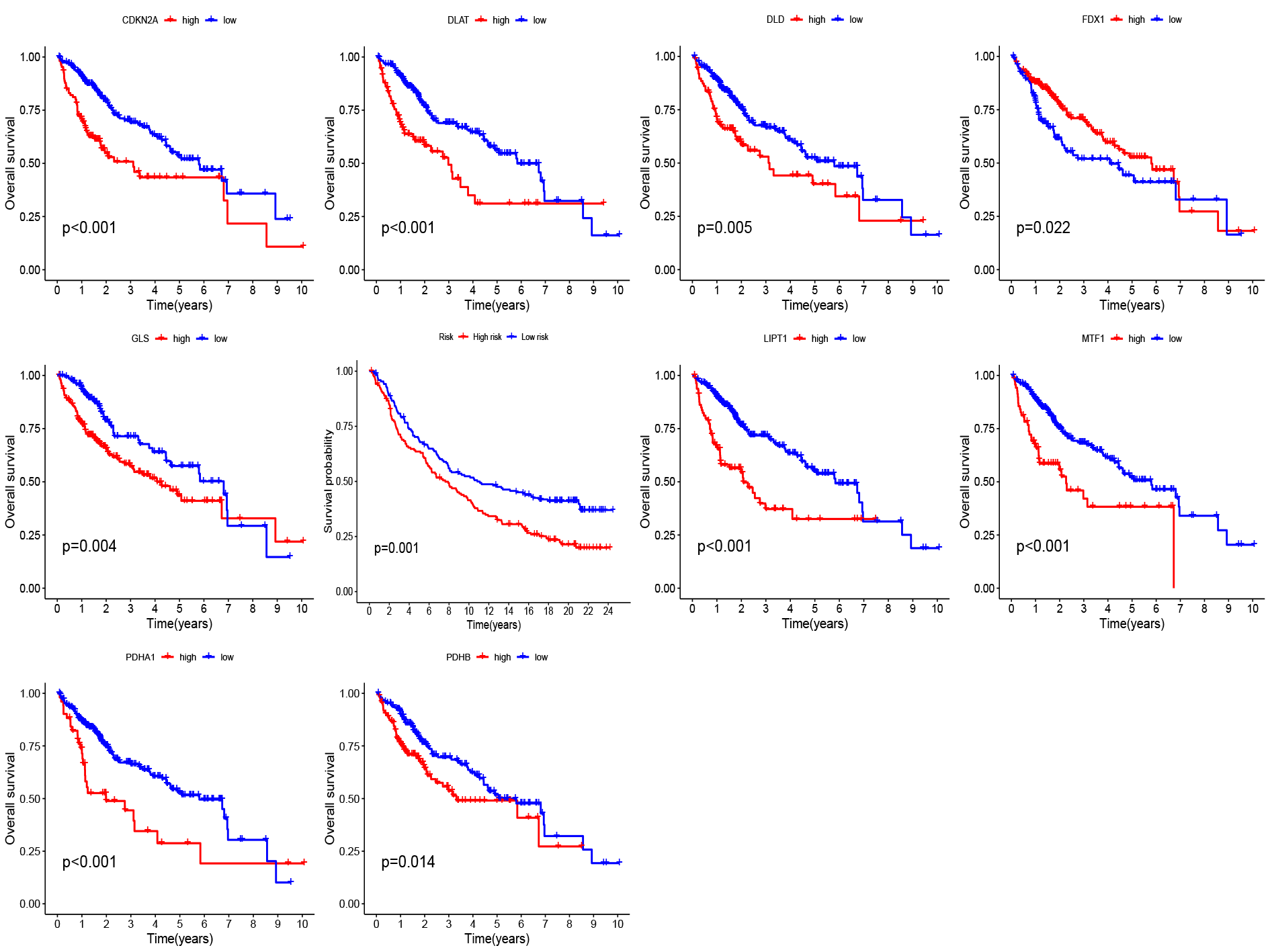

Supplement: Supplementary file 1 [file Image2.TIF]

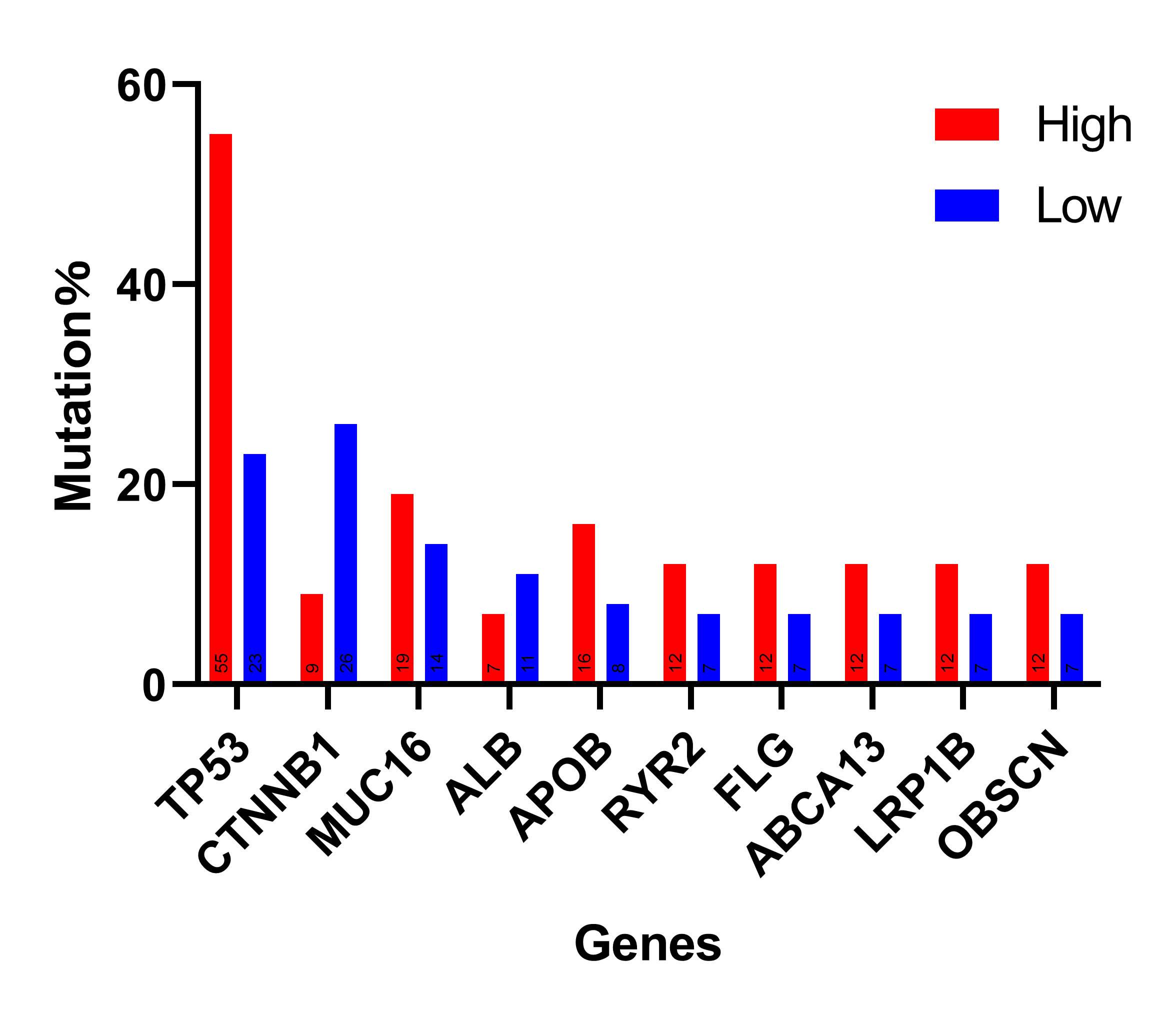

Supplement: Supplementary file 2 [file Image1.TIF]
